# Validity of Absolute Intake and Nutrient Density of Protein, Potassium, and Sodium Assessed by Various Dietary Assessment Methods: An Exploratory Study

**DOI:** 10.3390/nu12010109

**Published:** 2019-12-31

**Authors:** Laura Trijsburg, Anouk Geelen, Paul J.M. Hulshof, Pieter van’t Veer, Hendriek C. Boshuizen, Peter C.H. Hollman, Gertjan van Dijk, Edith J.M. Feskens, Jeanne H.M. de Vries

**Affiliations:** 1 Division of Human Nutrition and Health, Wageningen University, 6700 AA Wageningen, Gelderland, The Netherlands; anouk.geelen@wur.nl (A.G.); pieter.vantveer@wur.nl (P.v.V.); hendriek.boshuizen@wur.nl (H.C.B.);; 2 Wageningen Plant Research, Biometris, Wageningen University, 6700 AA Wageningen, Gelderland, The Netherlands; 3 Groningen Institute for Evolutionary Life Sciences (GELIFES), Unit Behavioral Neuroscience and ESRIG Center for Isotope Research, University of Groningen, 9747 AG Groningen, The Netherlands; gertjan.van.dijk@rug.nl

**Keywords:** dietary assessment, doubly labeled water, measurement errors, multivariate models, nutrient density, validation

## Abstract

It is suggested that nutrient densities are less affected by measurement errors than absolute intake estimates of dietary exposure. We compared the validity of absolute intakes and densities of protein (kJ from protein/total energy (kJ)), potassium, and sodium (potassium or sodium (in mg)/total energy (kJ)) assessed by different dietary assessment methods. For 69 Dutch subjects, two duplicate portions (DPs), five to fifteen 24-h dietary recalls (24 hRs, telephone-based and web-based) and two food frequency questionnaires (FFQs) were collected and compared to duplicate urinary biomarkers and one or two doubly labelled water measurements. Multivariate measurement error models were used to estimate validity coefficients (VCs) and attenuation factors (AFs). This research showed that group bias diminished for protein and sodium densities assessed by all methods as compared to the respective absolute intakes, but not for those of potassium. However, the VCs and AFs for the nutrient densities did not improve compared to absolute intakes for all four methods; except for the AF of sodium density (0.71) or the FFQ which was better than that of the absolute sodium intake (0.51). Thus, using nutrient densities rather than absolute intakes does not necessarily improve the performance of the DP, FFQ, or 24 hR.

## 1. Introduction

In nutritional epidemiology, it is common practice to focus on the variation in dietary composition, by using either energy adjustment or nutrient densities [1]. Using densities reduces between person variation due to extraneous factors (which are not confounders) such as differences in body composition [1]. Moreover, the impact of measurement errors on estimates of dietary exposure may be reduced, thereby strengthening the observed diet–disease associations [2]. In the OPEN-study, protein densities instead of absolute intakes estimated by 24-h dietary recalls (24 hRs) and food frequency questionnaires (FFQs) were indeed less affected by measurement errors [3]. On the other hand, other evidence showed a weakening of the observed diet–disease association based on nutrient densities as compared to absolute intakes [4]. These differences found could be ascribed to substantial measurement error in the estimated energy intake [5]. To objectively evaluate energy intakes, the doubly labelled water (DLW) technique should be used under the assumption of a stable body weight of the subjects [6].

A common method to express dietary composition is by nutrient densities, where the energy intake derived from the nutrient, or the absolute amounts consumed (for non-energy bearing nutrients) is divided by total energy intake. Nutrient densities can be calculated directly from the data on the individual level [1]. A pooling of 5 American validation studies, including the before mentioned OPEN study, showed that protein, potassium and sodium densities were less affected by measurement error compared to the absolute nutrient intakes estimated by the FFQs but this was not so pronounced for 24 hRs [7,8]. In the present study, we compared the validity of nutrient densities and absolute intakes of protein, potassium, and sodium estimated by four dietary assessment methods: FFQ, telephone-based 24 hR (24 hRT), web-based 24 hR (24 hRW) and duplicate portion (DP). As reference methods we used the respective recovery biomarkers of these nutrients and DLW for the intake of energy.

## 2. Materials and Methods

### 2.1. Study Participants and Design

The study set-up has previously been described [9]. In short: 200 participants of DuPLO, a Dutch validation study which is part of the NQplus study, were invited by email to have their energy expenditure assessed by DLW. Participants following a weight loss diet at the time of this study, using diuretics, being pregnant, suffering from heart or kidney failure or malabsorption were not enrolled in this sub-study. Recruitment was stopped after reaching the targeted sample size of 70 participants. Furthermore, 30 of them completed a second energy expenditure measurement by DLW (~5 months later). The participants, aged 20–70 years, lived in Wageningen and surroundings, the Netherlands. Baseline measurements included a physical examination, and general and lifestyle questionnaires (including questions about health and education). Within a timeframe of 1.5 years, participants collected two DPs (~5 months apart), two 24-h urines (~1 year apart), completed zero to nine (average 6) 24 hRW (~3 months apart) and zero to eight (average 5) 24 hRT (~4 months apart), and filled out two FFQs (~7 months apart). The number of 24 hRT and 24 hRW per subject varied because part of the participants was enrolled in another sub-study of the NQplus study in which larger numbers of 24 hRs were collected. In the DuPLO study we used all available 24 hR data from the NQplus study. As for all methods the time between replicates varied per person, we report the average time between the replicates. Participants with DLW data but missing data for one or more of the other methods were included in the data analysis. Written informed consent was obtained from every participant. The study proposal was approved by the institutional review board of Wageningen University. Investigations were carried out following the rules of the Declaration of Helsinki of 1975 revised in 2013.

### 2.2. Dietary Assessment by 24-h Dietary Recall and Food Frequency Questionnaire

Both the telephone and web-based 24 hR assessments followed a standardized protocol according to the 5-step multiple pass method [10]. For the 24 hRW, participants received an unannounced email invitation to fill out the 24 hRW in the web-based program Compl-eat, about their intake of the day before. If participants did not complete the 24 hRW within 24 h a new invitation was sent within three to ten days. For the 24 hRT, participants got an unannounced phone call from a trained dietician. Portion sizes for 24 hRW and 24 hRT were reported using household measures, standard portion sizes, weight in grams, or volume in liters [11].

A previously validated 180 item FFQ [12,13] was administered via the web using the online open-source survey tool Limesurvey^TM^. The reference period for the FFQ was one month and standardized household measures were used to assess portion sizes [11].

Self-reported dietary intake data from 24 hRW, 24 hRT, and FFQ were converted into energy and nutrient data using the Dutch food composition database of 2011 [14].

### 2.3. Duplicate Portion and 24-h Urine Collection

Participants received verbal and written instructions preceding the collection of the DP and 24-h urine. For the DP the participants collected an identical portion of all edible foods and drinks consumed over a 24-h period in collection baskets and stored them in a cool box (5 °C). At the study center, DPs were weighed, homogenized in a blender (Waring Commercial model 34BL22) and 2.5 mL 0.02% tert-butylhydroquinon in ethanol was added per kg of DP as antioxidant. Samples were stored within 1 h at −20 °C until further analysis. Part of the sample was freeze dried before analysis.

The 24-h urine collection started after discarding the first voiding on the morning of the collection day and included the first voiding on the morning of the next day. The preservative lithium dihydrogenphosphate (25 g) was added to the collection containers. Subjects were instructed to ingest in total three 80 mg para-aminobenzoic acid (PABA) tablets (PABA check, Elsie Widdowson Laboratory, Cambridge, UK) during breakfast, lunch and dinner on the day of collection to check for completeness of the urine collection. At the study center, the urine collections were mixed, weighted, aliquoted and stored at −20 °C until further analyses.

### 2.4. Laboratory Analysis

Potassium and sodium in urine were analyzed by ion selective electrode (Roche 917 analyzer, Indianapolis, IN, USA) and the intake was calculated taking into account extra-renal and fecal losses of 19% for potassium [15] and 14% for sodium [16]. Potassium and sodium in the DP were assessed by inductively coupled plasma atomic emission spectroscopy (ICP-AES, Varian Australia Pty Ltd., Mulgrave, Australia; ISO, 2010). Nitrogen was assessed with the Kjeldahl technique [17] in both DP and urine. The amount of protein was calculated using a nitrogen to protein conversion factor of 6.25 [18], and assuming an average ratio of urinary nitrogen excretion to dietary nitrogen of 0.81 [19]. The fat content of the DP was assessed by the acid hydrolysis method [20], ash by heating the freeze dried food in a muffle furnace at 550 °C [21], alcohol by gas chromatography [22], and the moisture content was assessed by drying in a vacuum oven [21]. PABA in urine was analyzed by HPLC [23]. See Supplementary Material for quality control measures.

We assumed moisture, ash, fat, protein, alcohol, and total carbohydrates (including dietary fiber) to sum up to 100% of the total weight of the DPs [24]. Total carbohydrates were calculated by difference [25]. Energy content of the DPs was subsequently calculated from the total amount of protein, fat, total carbohydrates and alcohol using the general Atwater factors for these nutrients: 17, 37, 17, and 29 kJ per gram respectively.

### 2.5. Energy Expenditure Measured by Doubly Labeled Water

Total energy expenditure for each participant covering an eleven day period was assessed by DLW method using the two-point protocol [26]. In the morning on the first day of the DLW period, body weight and height of each subject were measured to the nearest 0.1 kg and 0.1 cm, respectively. Next, baseline urine and saliva samples were collected followed by ingestion of a dose of DLW. Saliva samples were collected as back up samples if urine samples would not be sufficient or generate invalid results. Subjects received a mixture of 1.8 g 10% enriched H_2_^18^O (Centre for Molecular Research Ltd., Moscow, Russia) and 0.12 g 99.8% enriched ^2^H_2_O (Cambridge Isotope Laboratories, Inc, Andover, MA, USA) per kg body water. It was assumed that body weight of males and females comprised 55% and 50% body water respectively [27]. Additional urine and saliva samples were collected three and four hours post dose. Eleven days after the dosing of the isotopes, subjects revisited the study center at the same time as the three hours post dose collection on day 1. Body weight was re-measured and two urine and saliva samples were collected with an interval of one hour. Isotopic enrichment of the samples and diluted doses were analyzed at the Center for Isotope Research, Groningen, The Netherlands as described elsewhere [28]. Enrichments expressed as delta units were converted into parts per million excess [26,29]. ^2^H and ^18^O dilution spaces were calculated from the plateau enrichments at three and four hours post dose. Total body water was calculated as the average of the ^2^H dilution space divided by 1.041 and ^18^O dilution space divided by 1.01 to account for non-aqueous isotope exchange [30]. The rate of carbon dioxide production was calculated by the equation proposed by Schoeller et al. [31]. Total energy expenditure was calculated using the modified Weir equation [32] assuming a respiratory quotient of 0.85. See Supplementary Material for quality control measures. 

### 2.6. Measurement Error Model

In the measurement error model it was assumed that protein, sodium, and potassium intake assessed by urinary excretion and energy expenditure assessed by DLW were unbiased estimates of true intake [33]. A linear relationship between dietary intake assessed by DP, 24 hRT, 24 hRW, FFQ, or biomarker with the true (unknown) intake *T* was assumed. In the measurement error model, *i* indicates the person and *j* the occasion; *α_X_* the constant bias; *β_X_* proportional scaling bias; *w_xi_* person specific bias (psb); *ε_xij_* the random within person error with mean zero and constant variance for method X and *ε_Mij_* similarly for the biomarker. Replicates contributed to the estimation of within person random error. Method X is either: DP, 24 hRT, 24 hRW, or FFQ.

(1) $$\mathrm{Biomarker}:\;Mij=T+\varepsilon_{Mij}$$

(2) $$\mathrm{Method}\;X:\;Xij=\alpha_{X}+\beta_{X}T+w_{Xi}+\varepsilon_{Xij}$$

### 2.7. Statistical Analysis

Data of one participant were excluded because of physiologically implausible body water changes between repeated measurements while body weight remained stable. Thus data of 69 participants (37 men, 32 women) were included for analysis, 29 of them had duplicate measurements (16 men, 13 women). Descriptive statistics are presented as mean (SD) or percentages. Protein densities were calculated by dividing the energy provided by protein (1 g protein = 17 kJ) by total energy (in kJ). For potassium and sodium we used the ratio of the total amount of the nutrient (in mg) to total energy (in kJ). For the denominator of the biomarker densities we used the average energy expenditure per person from DLW if two measurements were available; otherwise the single DLW estimate was used (40 subjects). Using the average of two DLW measurements caused unwanted correlations between densities at the two time points. A sensitivity analysis, where densities for the urinary biomarkers at baseline were calculated with the first DLW measurement and for participants with a second DLW measurement at year one with the second DLW measurement, did not substantially affect the model outcomes. We therefore report the data using one or the average of two DLW measurements. Visual inspection of QQ-plots of the data did not show evidence of non-normality. Outliers for the dietary intake assessments were checked but as there was no indication for incorrectly recording of these data, they were maintained in the dataset for further analysis. The validity coefficient (VC, *ρ_XT_*, Formula (3)) was used to assess the loss of statistical power to detect a diet–disease association and the ability to rank participants according to their intake, whereas the attenuation factor (AF, *λ_x_*, Formula (4)) provides information about the extent to which diet–disease associations are affected by measurement error:

(3) $$\rho_{XT}=\sqrt{\frac{{\beta_{X}}^{2}\;varT}{{\beta_{X}}^{2}\;varT+\frac{var\;\varepsilon_{Xij}}{k}+varw_{Xi}}}$$

(4) $$\lambda_{X}=\frac{{\rho_{XT}}^{2}}{\beta_{X}}$$

where *varT* is the variance of the true nutrient intake, *varε_Xij_* the variance of the random within person error, *varw_Xi_* the variance of the psb and *k* the number of replicates of the method. We assessed the theoretical case of obtaining an infinite (∞) number of measurements, in which within person variation cancels out from the equation.

To understand observed differences in VCs and AFs between methods, the size of the different variances of psb and random within person error and the proportional scaling bias are relevant. To facilitate comparisons between methods and between different nutrients, we expressed the variances of the errors for all nutrients relative to the estimate of the variance of the true intake [3]:

(5) $$\mathrm{Random}\;\mathrm{error}\;\mathrm{variance}\;\mathrm{ratio}:\;\frac{var\;\varepsilon_{Xij}}{varT}$$

(6) $$\mathrm{Person}\;\mathrm{specific}\;\mathrm{bias}\;\mathrm{variance}\;\mathrm{ratio}:\;\frac{varw_{Xi}}{varT}$$

(7) $$\mathrm{Combined}\;\mathrm{error}\;\mathrm{variance}\;\mathrm{ratio}:\;\frac{var\;\varepsilon_{Xij}+\;varw_{Xi}}{varT}$$

Using the proposed minimum 78% PABA recovery as a cut-off point for complete urine collection [23], N = 19 (14%) of the urines were judged incomplete. A sensitivity analysis in which urines with <78% PABA recovery were excluded did not substantially change the model outcomes. This was in line with findings of Subar et al. who observed a modest effect on correction factors when urines were excluded based on PABA recovery [34]. We therefore report the results based on the complete urine set. For all statistical tests SAS version 9.3 (SAS Institute Inc. Cary, NC, USA, 2012) was used.

## 3. Results

At baseline, participants (N = 69) were on average 57.3 (SD 9.1) years old, had a mean body mass index of 25.5 (SD 3.6) kg/m^2^ and 20.3% was classified as lower educated (primary or lower education) while 47.8% was classified as highly educated (university or college degree).

On average, all four dietary assessment methods underestimated energy and nutrient intakes as compared to the biomarkers. Energy intake was underestimated by on average 20% (between methods range 16.1–21.8%) as compared to the energy expenditure measured by DLW (Table 1). Compared to their respective urinary biomarkers, protein intake was underestimated to a comparable extent (between methods range 10.4–23.0%) while sodium was seriously underestimated (between methods range 27.3–41.3%). The percentage bias for protein and sodium densities was smaller than that for their respective absolute intakes. In contrast, bias in percentage for potassium densities was larger than for absolute potassium intake.

For energy, the VC for a single FFQ measurement (0.63) was comparable to that based on three measurements for the DP (0.59, Table 2). The VC for energy based on three 24 hRT was only 0.14 while it was 0.48 for three 24 hRW. The AF for energy roughly followed a similar pattern: For a single FFQ measurement it was 0.51, it was higher for three replicates of the DP (0.69, Table 3), and considerably lower for three 24 hRT (0.17) or 24 hRW (0.40).

In Table 2 and Table 3 VCs and AFs were compared between nutrient densities and absolute nutrient intakes for single measurements. Increasing the number of measurements (up to infinite), showed a comparable pattern as described for single measurements. VCs for protein densities were lower than for absolute intakes for the DP (0.28 vs. 0.70, Table 2) and FFQ (0.37 vs. 0.70) whereas for the 24 hRT and 24 hRW VCs for protein densities and absolute protein intake were comparable. For potassium, VCs were lower for the densities than for absolute potassium intake for all four methods. For sodium, VCs for densities and absolute intake were comparable for all methods except for the 24 hRW, for which lower estimates for sodium density than for absolute sodium intake were observed (0.22 and 0.49 respectively).

Comparing AFs for the same method between protein densities and absolute protein intakes showed comparable estimates, except for the DP for which lower estimates for protein densities than for absolute protein intake were observed (0.30 and 0.78 respectively, Table 3). AFs for potassium densities for the DP, 24 hRT and 24 hRW were lower than for absolute potassium intakes while they were comparable for the FFQ. AFs for sodium density and absolute sodium intake were comparable for both DP and 24 hRT, whereas for the 24 hRW, a lower AF for sodium density (0.15) than for absolute intake (0.35) was seen. In contrast, for the FFQ, a higher AF for sodium density (0.71) than for absolute intake (0.51) was observed.

Table 4 shows the variances of the error components relative to the variance of the true intake. A lower ratio means the estimated intakes were less affected by random within person error, psb, or combined error (the sum of the variances of random within person error and psb). Consistent with the concept of the methods, the combined error variance ratio for energy intake was highest for the 24 hRW (2.60, Table 4) due to the high random error variance ratio (2.19). The FFQ had the highest psb variance ratio (0.76).

For DP and 24 hRT, combined error variance ratios were higher for the nutrient densities than for the absolute intakes. For the FFQ, combined error variance ratios were lower for the nutrient densities than for the absolute intakes: For protein (0.44 vs. 0.58, Table 4), potassium (0.70 vs. 1.05), and sodium (0.25 vs. 0.65), which can be largely attributed to the lower psb variance ratios for the nutrient densities. For the 24 hRW, the combined error variance ratio for sodium density was higher, while for protein density it was slightly lower and for potassium it was lower (2.48 vs. 3.03) than for the respective absolute intakes. The latter was due to the diminished random error variance ratio (from 2.92 to 1.87).

Proportional scaling bias, indicated by β_X_ in Equation (2), was less if its value was closer to 1. Proportional scaling bias influenced the energy intake assessed by the 24 hRT to a major extent (0.11, Table 4), whereas the FFQ was least influenced (0.78). Overall, proportional scaling biases affected the nutrient densities for all dietary assessment methods to a larger extent than the absolute nutrient intakes. However, absolute sodium intake and sodium density had comparable proportional scaling biases for DP (0.56 and 0.60) and 24 hRT (both 0.21).

## 4. Discussion

In our study, the DP, both 24 hRs and FFQ showed comparable patterns for bias on the group level: Bias of protein and sodium densities was less than that of the absolute intakes, whereas bias of potassium density was larger than that of absolute potassium intake. The VCs and AFs for DP, both 24 hRs and FFQ did not improve for nutrient densities compared to absolute intakes of protein, sodium, and potassium, except for the AF of the FFQ for sodium. For potassium, densities performed less than absolute intakes, but also for protein and sodium this was seen for some of the VCs and AFs. Proportional scaling bias, random within person error and psb, all affected protein, potassium and sodium density estimates to a larger extent than their absolute nutrient intakes. Exceptions to this observation were seen for the FFQ, where the psb was smaller for all nutrient densities than for the absolute intakes, and for the 24 hRW, where the random error for potassium density was smaller than for absolute potassium intake.

VCs and AFs of energy intake were highest for the FFQ and DP, followed by the 24 hRW and least for the 24 hRT. The poor validity for the 24 hRT is consistent with findings from an American pooling project [7]. It appeared partly due to a large proportional scaling bias (0.11). Although an explanation for the latter is lacking, it is clear that the errors in estimated energy intake carry forward to the estimated densities, most seriously for the 24 hRT followed by the 24 hRW and least for DP and FFQ.

Comparing our findings with an American pooling project showed that our finding of the higher group level bias of potassium density compared to potassium was similar. However, they did not observe the consistent improvement of group level bias for protein and sodium densities as compared to absolute intakes. When we compare the VCs and AFs of the nutrient densities and absolute nutrient intakes our results were not in line with their findings either. They observed that VCs of FFQs improved for nutrient densities compared to absolute nutrient intakes, where for the 24 hR, the VCs were rather comparable [7,8]. In our study the VCs did not show an improvement for any of the dietary assessment methods for the nutrient densities, and especially for potassium density, they worsened compared to the absolute intakes. In the Pooling project the AFs improved for both the FFQ and 24 hR for the nutrient densities compared to the absolute intakes [7,8]. We only observed an improvement of the AF for sodium density for the FFQ and especially for potassium density, AFs worsened compared to the absolute intakes. Our VCs and AFs for the absolute intakes generally tended to be higher than those observed in the Pooling project, where our VCs and AFs of the nutrient densities were of similar magnitude. Since our absolute nutrient intake already had a relatively high validity there was not much room for improvement of validity when using nutrient densities. Differences in validity between the studies were to be expected as the dietary assessment methods were not exactly the same, and also the dietary pattern of our population differed from that in the pooling project. Unfortunately, inference on such issues is limited by the precision of our estimates, because of the smaller sample size of our study.

Looking at the practical implications of our findings let us take the example of potassium vs. potassium density for the DP. A higher bias (either over- or underestimation) will render less accurate results and affect conclusions on for example prevalence of nutrient adequacy in the population. In our example the potassium density (with an average overestimation of 21.7%) will wrongly classify a high percentage of people as nutrient adequate, whereas for absolute potassium intake an underestimation of nutrient adequacy will be found. VCs mainly provide information on the loss of statistical power to detect a diet disease association. Given a VC of 0.66 for potassium, instead of a study sample of 100, a study sample of 230 (100/(0.66^2^)) will have to be recruited whereas for potassium density the recruited sample will have to be 1041 (100/(0.31^2^)). AFs provide information on the extent to which relative risks (RR) or odds ratios are affected by measurement error. Given a true RR of 2 the observed RR in this study, with an AF for potassium of 0.45, will be 1.3 (2^0.45^) and 1.2 (2^0.25^) for potassium density. Which outcome, bias, VC or AF, is of most importance depends on the aim of the study. We observed that for all absolute nutrient intakes for the FFQ the psb variance ratios were larger than for the DP and 24 hRs. This might be due to the specific methodological characteristics of the FFQ: Grouping of foods into a smaller number of food items limits the freedom to report specific single foods which increases the person specific bias variance, especially when comparing to open ended dietary assessment methods that allow much more specificity at the food level (DP and 24 hRs).

The combined error variance ratios for our FFQ for all nutrient densities were smaller than for the absolute intakes. Michels et al. observed that error correlations between nutrients and energy for a FFQ were larger than those for a food record [35]. As error correlations between nutrient and energy intake partially cancel out when using densities [2,36], this might explain the smaller combined error variance ratios for our FFQs for the nutrient densities compared to the absolute intakes. However this did not improve the VCs and AFs for the nutrient densities, as the proportional scaling bias was larger for the nutrient densities than the absolute nutrient intakes.

The different methods did not exactly cover the same time period. However, our interest was to evaluate the validity of a person’s usual dietary intake not of the dietary intake on a specific day. We assumed energy and nutrient intakes of a person to be fairly stable over the 1.5 year in which the person’s measurements were taken. Thus although intake data measured by the different dietary assessment methods did not cover the same time period, the estimates were considered to represent a person’s usual energy and nutrient intakes.

We found that accounting for energy by means of energy densities does not necessarily diminish the impact of measurement errors on estimates of dietary exposure. These results serve to highlight, that validation studies should be incorporated in the study design, irrespective of whether absolute dietary intake or nutrient densities are the measure of interest of dietary exposure in nutritional epidemiology.

## 5. Conclusions

From this study it can be concluded that in this rather small, highly educated Dutch population, expressing diet in terms of nutrient densities rather than absolute intakes did not improve the performance of the assessment methods for protein, potassium and sodium.

## Figures and Tables

**Table 1:** Mean intake and bias of energy, protein, potassium, sodium, and their nutrient densities for the biomarker, DP, 24 hRT, 24 hRW and FFQ.

	Energy (MJ)	Protein (g)	Protein Density ^a^	Potassium (mg)	Potassium Density ^b^	Sodium (mg)	Sodium Density ^b^
Biomarker							
Mean(SD)	11.4(2.5)	101.5(27.1)	0.15(0.03)	3888(886)	0.35(0.09)	4069(1263)	0.36(0.12)
DP							
Mean(SD)	8.9(2.0)	77.3(19.5)	0.15(0.03)	3554(868)	0.41(0.08)	2580(853)	0.29(0.09)
Bias ^c,d^ (%)	−19.7(17.2)	−21.4(19.3)	0.87(24.8)	−6.4(2.3)	21.7(34.3)	−34.3(18.8)	−16.0(25.5)
24 hRT							
Mean(SD)	9.0(1.7)	83.2(19.4)	0.16(0.02)	3506(691)	0.40(0.08)	2646(674)	0.30(0.06)
Bias ^c,d^ (%)	−16.1(20.5)	−10.4(20.3)	9.7(22.0)	−4.9(19.2)	18.9(25.7)	−27.3(27.7)	−10.4(34)
24 hRW							
Mean(SD)	8.8(2.4)	82.2(25.1)	0.16(0.03)	3429(1005)	0.40(0.08)	2663(832)	0.31(0.07)
Bias ^c,d^ (%)	−20.6(21.2)	−16.5(23.0)	7.8(21.3)	−10.4(20.3)	20.1(30.4)	−30.1(25.6)	−7.5(32.3)
FFQ							
Mean(SD)	8.8(2.6)	76.4(25.6)	0.15(0.02)	3466(1018)	0.40(0.06)	2242(833)	0.25(0.04)
Bias ^c,d^ (%)	−21.8(19.0)	−23.0(22.5)	−0.25(20.8)	−9.5(21.7)	19.2(27.5)	−41.3(23.8)	−25.1(23.1)

Abbreviations: DP, duplicate portion; FFQ, food frequency questionnaire; SD, standard deviation; 24 hRT, 24-h dietary recall telephone-based; 24 hRW, 24-h dietary recall web-based ^a^ kJ from protein divided by total energy in kJ ^b^ mg potassium or sodium divided by total energy in kJ ^c^ % bias was calculated on the individual level using the biomarker as the true intake and then averaged ^d^ Mean (SD).

**Table 2:** Validity coefficients (VC) of energy, protein, potassium, sodium and their nutrient densities for the DP, 24 hRT, 24 hRW and FFQ with the biomarker as the Reference method (mean (SE)).

	k	Energy	Protein	Protein Density	Potassium	Potassium Density	Sodium	Sodium Density
DP	1	0.49(0.12)	0.70(0.07)	0.28(0.11)	0.66(0.10)	0.31(0.12)	0.53(0.09)	0.48(0.09)
	2	0.56(0.14)	0.77(0.07)	0.32(0.13)	0.76(0.11)	0.36(0.14)	0.66(0.09)	0.61(0.10)
	3	0.59(0.14)	0.80(0.07)	0.34(0.13)	0.81(0.12)	0.39(0.15)	0.73(0.08)	0.69(0.09)
	∞	0.68(0.16)	0.87(0.07)	0.39(0.15)	0.93(0.14)	0.46(0.18)	1.00 ^a^	1.00 ^a^
24 hRT	1	0.10(0.15)	0.45(0.12)	0.34(0.08)	0.56(0.09)	0.37(0.09)	0.18(0.12)	0.15(0.08)
	2	0.13(0.18)	0.54(0.13)	0.43(0.09)	0.68(0.10)	0.45(0.11)	0.23(0.16)	0.20(0.11)
	3	0.14(0.20)	0.58(0.13)	0.48(0.10)	0.74(0.11)	0.50(0.12)	0.26(0.18)	0.24(0.14)
	∞	0.18(0.25)	0.71(0.14)	0.71(0.14)	0.94(0.13)	0.65(0.15)	0.42(0.28)	0.66(0.41)
24 hRW	1	0.34(0.09)	0.44(0.08)	0.31(0.07)	0.57(0.08)	0.21(0.09)	0.49(0.11)	0.22(0.09)
	2	0.43(0.12)	0.54(0.09)	0.40(0.09)	0.70(0.09)	0.27(0.12)	0.61(0.14)	0.29(0.12)
	3	0.48(0.12)	0.59(0.10)	0.45(0.10)	0.76(0.10)	0.30(0.13)	0.67(0.14)	0.34(0.13)
	∞	0.68(0.15)	0.75(0.10)	0.63(0.13)	0.97(0.12)	0.41(0.17)	0.92(0.17)	0.63(0.24)
FFQ	1	0.63(0.11)	0.70(0.08)	0.37(0.11)	0.73(0.11)	0.41(0.13)	0.46(0.16)	0.38(0.15)
	2	0.65(0.11)	0.72(0.08)	0.40(0.12)	0.76(0.11)	0.44(0.14)	0.48(0.17)	0.42(0.16)
	3	0.65(0.11)	0.73(0.08)	0.42(0.12)	0.76(0.11)	0.45(0.14)	0.48(0.17)	0.43(0.16)
	∞	0.67(0.11)	0.74(0.08)	0.45(0.13)	0.78(0.12)	0.47(0.14)	0.50(0.17)	0.47(0.17)

Abbreviations: DP, duplicate portion; FFQ, food frequency questionnaire; k, number of measurements; SE, standard error; 24 hRT, 24-h dietary recall telephone-based; 24 hRW, 24-h dietary recall web-based All measurement error models were adjusted for BMI and gender ^a^ No person specific bias was observed; ∞, infinite.

**Table 3:** Attenuation factors (AF) of energy, protein, potassium, sodium, and their nutrient densities for the DP, 24 hRT, 24 hRW and FFQ with the biomarker as the reference method (mean(SE)).

	k	Energy	Protein	Protein Density	Potassium	Potassium Density	Sodium	Sodium Density
DP	1	0.46(0.12)	0.78(0.10)	0.30(0.12)	0.45(0.08)	0.25(0.10)	0.50(0.11)	0.39(0.10)
	2	0.61(0.15)	0.96(0.11)	0.39(0.16)	0.60(0.10)	0.34(0.14)	0.78(0.14)	0.63(0.13)
	3	0.69(0.16)	1.03(0.12)	0.44(0.18)	0.67(0.12)	0.39(0.16)	0.96(0.15)	0.79(0.14)
	∞	0.90(0.23)	1.23(0.17)	0.57(0.24)	0.89(0.19)	0.56(0.24)	1.80(0.41)	1.67(0.44)
24 hRT	1	0.09(0.13)	0.42(0.11)	0.27(0.07)	0.40(0.08)	0.23(0.07)	0.15(0.10)	0.11(0.06)
	2	0.14(0.20)	0.60(0.14)	0.44(0.11)	0.58(0.10)	0.35(0.09)	0.25(0.17)	0.20(0.11)
	3	0.17(0.24)	0.70(0.15)	0.56(0.13)	0.69(0.11)	0.43(0.11)	0.33(0.22)	0.29(0.16)
	∞	0.29(0.40)	1.04(0.21)	1.21(0.33)	1.11(0.21)	0.73(0.20)	0.84(0.58)	2.09(1.96)
24 hRW	1	0.20(0.06)	0.31(0.07)	0.24(0.06)	0.27(0.05)	0.13(0.06)	0.35(0.08)	0.15(0.06)
	2	0.32(0.08)	0.46(0.08)	0.39(0.10)	0.40(0.06)	0.21(0.09)	0.54(0.12)	0.27(0.11)
	3	0.40(0.10)	0.55(0.09)	0.49(0.12)	0.48(0.07)	0.26(0.11)	0.67(0.14)	0.36(0.14)
	∞	0.78(0.19)	0.88(0.15)	0.99(0.25)	0.77(0.13)	0.47(0.21)	1.25(0.26)	1.23(0.56)
FFQ	1	0.51(0.09)	0.65(0.09)	0.52(0.17)	0.49(0.09)	0.45(0.15)	0.51(0.18)	0.71(0.28)
	2	0.54(0.10)	0.69(0.09)	0.62(0.19)	0.52(0.09)	0.50(0.17)	0.54(0.20)	0.85(0.33)
	3	0.55(0.10)	0.70(0.09)	0.66(0.21)	0.53(0.09)	0.53(0.17)	0.56(0.20)	0.90(0.35)
	∞	0.57(0.10)	0.72(0.10)	0.75(0.24)	0.55(0.10)	0.57(0.19)	0.59(0.21)	1.04(0.41)

Abbreviations: DP, duplicate portion; FFQ, food frequency questionnaire; k, number of measurements; SE, standard error; 24 hRT, 24-h dietary recall telephone-based; 24 hRW, 24-h dietary recall web-based All measurement error models were adjusted for BMI and gender; ∞, infinite.

**Table 4:** Error ratios and proportional scaling biases for energy, protein, potassium, sodium, and their nutrient densities for the DP, 24 hRT, 24 hRW, and FFQ with the biomarker as the reference method (mean(SE)).

	Energy	Protein	Protein Density	Potassium	Potassium Density	Sodium	Sodium Density
DP							
Combined ratio ^a^	0.84(0.23)	0.41(0.10)	0.81(0.20)	1.21(0.40)	1.40(0.43)	0.81(0.27)	1.19(0.42)
Random ratio ^b^	0.53(0.16)	0.29(0.08)	0.43(0.12)	1.06(0.39)	0.86(0.28)	0.81(0.27)	1.19(0.42)
Psb ratio^c^	0.30(0.14)	0.12(0.07)	0.39(0.14)	0.15(0.27)	0.54(0.24)	^d^	^d^
Prop scaling bias ^e^	0.51(0.16)	0.62(0.10)	0.26(0.11)	0.97(0.25)	0.38(0.17)	0.56(0.13)	0.60(0.16)
24 hRT							
Combined ratio ^a^	1.21(0.31)	0.91(0.20)	1.39(0.32)	1.38(0.43)	2.15(0.63)	1.39(0.51)	1.91(0.71)
Random ratio ^b^	0.83(0.21)	0.68(0.15)	1.22(0.28)	1.30(0.43)	1.68(0.50)	1.19(0.44)	1.86(0.70)
Psb ratio ^c^	0.38(0.14)	0.23(0.09)	0.17(0.09)	0.08(0.17)	0.46(0.22)	0.21(0.12)	0.06(0.09)
24 hRW							
Combined ratio ^a^	2.60(0.64)	1.63(0.35)	1.51(0.34)	3.03(0.92)	2.48(0.72)	1.50(0.52)	2.05(0.76)
Random ratio ^b^	2.19(0.55)	1.32(0.28)	1.26(0.28)	2.92(0.97)	1.87(0.55)	1.41(0.53)	1.89(0.71)
Psb ratio ^c^	0.41(0.18)	0.32(0.13)	0.25(0.10)	0.11(0.34)	0.61(0.24)	0.08(0.16)	0.16(0.11)
Prop scaling bias ^e^	0.59(0.18)	0.63(0.14)	0.41(0.10)	1.21(0.29)	0.35(0.16)	0.68(0.23)	0.32(0.15)
FFQ							
Combined ratio ^a^	0.92(0.27)	0.58(0.16)	0.44(0.11)	1.05(0.40)	0.70(0.22)	0.65(0.25)	0.25(0.09)
Random ratio ^b^	0.16(0.05)	0.11(0.03)	0.15(0.04)	0.26(0.10)	0.18(0.06)	0.11(0.04)	0.09(0.04)
Psb ratio ^c^	0.76(0.24)	0.47(0.14)	0.28(0.09)	0.79(0.36)	0.52(0.18)	0.54(0.22)	0.15(0.07)
Prop scaling bias ^e^	0.78(0.19)	0.75(0.14)	0.26(0.09)	1.11(0.29)	0.38(0.14)	0.42(0.19)	0.21(0.09)

Abbreviations: DP, duplicate portion; FFQ, food frequency questionnaire; Prop scaling bias, proportional scaling bias; Psb ratio, Person specific bias ratio; SE, standard error; 24 hRT, 24-h dietary recall telephone-based; 24 hRW, 24-h dietary recall web-based All measurement error models were adjusted for BMI and gender ^a^ Calculated according to Equation (7), ^b^ Calculated according to Equation (5), ^c^ calculated according to Equation (6), ^d^ No person specific bias was observed, ^e^ β_X_ in Equation (2).

## Supplementary Materials

The following are available online at https://www.mdpi.com/2072-6643/12/1/109/s1, S1 Laboratory quality control measures, Table S2: Validity Coefficients and Attenuation Factors after Log-transformation for Energy, Protein, Potassium, Sodium, and their Energy Densities for the DP, 24 hRT, 24 hRW and FFQ with the Biomarker as the Reference Method (Mean(SE)).
