# Supplementary material for: Validity of Absolute Intake and Nutrient Density of Protein, Potassium, and Sodium Assessed by Various Dietary Assessment Methods: An Exploratory Study
Source: Nutrients. 2019 Dec 31;12(1):109. doi: 10.3390/nu12010109 (PMC7019974; doi:10.3390/nu12010109)
Supplement: Supplementary file 1 [file nutrients-12-00109-s001.pdf]

S1 Laboratory quality control measures:

Within run coefficients of variation (CVw) and between run coefficients of variation (CVb) in urine were: CVw<1% and CVb<1% for both K and Na, for protein CVw=1.6% and CVb=1.3% and for PABA CVw=1.9% and CVb=1.3%. Participation in the External Quality Assessment Scheme of the Dutch Foundation for Quality Assessment in Medical Laboratories showed bias of -1.6% and +1.1% and analytical variation was 1.6% and 1.2% for urinary K and Na respectively. For the DPs quality control measures were as follows: protein CVw<1% and CVb<1%, K CVw<1% and CVb<1%, Na CVw=1.1% and CVb=1.7%, fat CVw = 0.9% and CVb=4.0%, ash CVw = 0.7% and CVb = 1.1% and for alcohol CVw = 4.3% and CVb=10.8%. For the DLW analysis of reference waters (biomedical enriched waters gravimetrically prepared from Vienna Standard Mean Ocean Water) showed analytical variations <0.5% for both isotopes and accuracy defined as deviation from the certified values were <1% for δ^{2}H and <0.3% for δ^{18}O. Isotope enrichment of ^{2}H and ^{18}O at 3 h and 4 h post dose differed on average 1.1% (range 0.0-4.5%) and 0.2% (range 0.0-1.1%) respectively. The ratio of deuterium dilution space to ^{18}O dilution space was on average 1.031 (range 1.000-1.073). Urine enrichments on the final day (day 11) were on average 44 ppm (range 24-65 ppm) above baseline for ^{2}H and 55ppm (range 27-90 ppm) for ^{18}O. Baseline values for ^{2}H and ^{18}O were 152 ppm (range 148-155 ppm) and 1994 ppm (range 1990-1998 ppm) respectively.

Table S1: Validity Coefficients and Attenuation Factors after Log-transformation for Energy, Protein, Potassium, Sodium and their Energy Densities for the DP, 24hRT, 24hRW and FFQ with the Biomarker as the Reference Method (Mean(SE))

		**DP**		**24hRT**		**24hRW**		**FFQ**	
	k[a]	ρ_{DPT}	λ_{DP}	ρ_{RTT}	λ_{RT}	ρ_{RWT}	λ_{RW}	ρ_{QT}	λ_{Q}
**Energy**	1	0.45(0.12)	0.32(0.09)	0.13(0.14)	0.09(0.09)	0.28(0.11)	0.15(0.06)	0.57(0.11)	0.38(0.08)
	2	0.51(0.13)	0.43(0.11)	0.16(0.16)	0.13(0.13)	0.34(0.12)	0.21(0.08)	0.60(0.11)	0.41(0.08)
	3	0.54(0.14)	0.48(0.12)	0.18(0.18)	0.16(0.16)	0.37(0.13)	0.25(0.09)	0.61(0.11)	0.42(0.09)
	∞	0.62(0.15)	0.63(0.17)	0.22(0.22)	0.25(0.24)	0.46(0.16)	0.39(0.14)	0.62(0.12)	0.45(0.09)
**Protein**	1	0.57(0.10)	0.52(0.09)	0.34(0.11)	0.26(0.09)	0.40(0.10)	0.24(0.06)	0.63(0.10)	0.48(0.08)
	2	0.64(0.10)	0.64(0.11)	0.42(0.13)	0.38(0.12)	0.47(0.11)	0.34(0.08)	0.65(0.10)	0.52(0.08)
	3	0.67(0.10)	0.70(0.12)	0.45(0.14)	0.45(0.14)	0.51(0.11)	0.40(0.09)	0.66(0.10)	0.53(0.09
	∞	0.74(0.10)	0.85(0.15)	0.57(0.16)	0.71(0.21)	0.62(0.13)	0.58(0.13)	0.68(0.10)	0.56(0.09)
**Protein**	1	0.30(0.11)	0.31(0.12)	0.37(0.08)	0.31(0.08)	0.35(0.07)	0.31(0.07)	0.41(0.11)	0.55(0.16)
**density**	2	0.35(0.13)	0.41(0.16)	0.46(0.09)	0.50(0.11)	0.45(0.09)	0.50(0.11)	0.44(0.12)	0.64(0.18)
	3	0.37(0.13)	0.46(0.18)	0.52(0.10)	0.63(0.14)	0.50(0.09)	0.62(0.13)	0.45(0.12)	0.68(0.19)
	∞	0.42(0.15)	0.61(0.24)	0.73(0.13)	1.26(0.31)	0.71(0.12)	1.25(0.29)	0.48(0.13)	0.77(0.22)
**Potassium**	1	0.64(0.12)	0.40(0.08)	0.55(0.11)	0.33(0.07)	0.62(0.10)	0.29(0.05)	0.76(0.13)	0.46(0.08)
	2	0.74(0.14)	0.55(0.11)	0.67(0.12)	0.48(0.10)	0.74(0.11)	0.41(0.07)	0.79(0.13)	0.50(0.09)
	3	0.79(0.14)	0.62(0.12)	0.73(0.13)	0.57(0.11)	0.80(0.12)	0.47(0.08)	0.80(0.13)	0.51(0.09)
	∞	0.92(0.17)	0.84(0.20)	0.92(0.16)	0.91(0.19)	0.96(0.14)	0.69(0.11)	0.83(0.14)	0.54(0.10)
**Potassium**	1	0.32(0.14)	0.27(0.12)	0.46(0.10)	0.33(0.08)	0.32(0.10)	0.21(0.07)	0.50(0.14)	0.56(0.16)
**density**	2	0.38(0.16)	0.38(0.16)	0.56(0.12)	0.50(0.12)	0.39(0.12)	0.32(0.11)	0.53(0.15)	0.64(0.18)

	3	0.40(0.17)	0.43(0.19)	0.62(0.13)	0.61(0.14)	0.43(0.14)	0.39(0.13)	0.55(0.15)	0.67(0.19)
	∞	0.48(0.20)	0.62(0.28)	0.80(0.16)	1.03(0.25)	0.57(0.17)	0.69(0.23)	0.57(0.15)	0.73(0.21)
**Sodium**	1	0.54(0.09)	0.33(0.08)	0.04(0.12)	0.02(0.06)	0.34(0.13)	0.15(0.06)	0.39(0.18)	0.25(0.11)
	2	0.67(0.09)	0.52(0.09)	0.05(0.16)	0.03(0.11)	0.43(0.15)	0.24(0.08)	0.41(0.18)	0.27(0.12)
	3	0.74(0.08)	0.63(0.10)	0.05(0.18)	0.04(0.14)	0.47(0.17)	0.29(0.10)	0.42(0.19)	0.28(0.13)
	∞	1.00[b]	1.14(0.27)	0.09(0.31)	0.12(0.39)	0.65(0.22)	0.54(0.18)	0.43(0.19)	0.30(0.13)
**Sodium density**	1	0.52(0.09)	0.34(0.08)	0.04(0.09)	0.02(0.05)	0.19(0.11)	0.09(0.05)	0.41(0.17)	0.44(0.18)
	2	0.65(0.09)	0.53(0.11)	0.05(0.13)	0.04(0.09)	0.25(0.14)	0.16(0.09)	0.44(0.18)	0.52(0.21)
	3	0.73(0.08)	0.66(0.13)	0.06(0.15)	0.05(0.13)	0.28(0.16)	0.21(0.12)	0.46(0.19)	0.55(0.23)
	∞	1.00[b]	1.25(0.33)	0.18(0.45)	0.46(1.23)	0.45(0.25)	0.53(0.30)	0.49(0.20)	0.63(0.26)

All measurement error models were adjusted for BMI and gender

[a]k = number of measurements

[b] No person specific bias was observed

∞ infinite
